# Redefining social medicine from Latin America: Historical foundations and contemporary directions

**DOI:** 10.1371/journal.pgph.0006213

**Published:** 2026-03-27

**Authors:** Francisco Ortega, Gabriel Abarca-Brown

**Affiliations:** 1 Catalan Institution for Research and Advanced Studies (ICREA), Barcelona, Spain; 2 Medical Anthropology Research Center, Universitat Rovira i Virgili, Tarragona, Spain; 3 Center for Culture and the Mind (CULTMIND), University of Copenhagen, Copenhagen, Denmark; 4 Diego Portales University, Santiago, Chile; Brigham and Women's Hospital, UNITED STATES OF AMERICA

## Abstract

This article examines the contemporary meaning of social medicine, a field marked by its porous boundaries, plurality, and contestation. Rather than offering a fixed definition, we trace its shifting forms across time, geography, and politics, positioning it as a “boundary object” that adapts to diverse contexts while retaining a minimal common identity. Comparative discussion with medical anthropology, social studies of medicine, global health, underscores social medicine’s distinct focus on structural determinants, inequities, and justice. We propose three elements that could be the basic common elements of social medicine, drawing on foundational tenets of Latin American Social Medicine for this classification: 1) political commitment to social justice, 2) the central role of social sciences, and 3) participatory methodologies rooted in community participation. We highlight how these elements informed transformative reforms while also noting how institutionalization sometimes diluted revolutionary impulses into bureaucratic logics. Finally, we analyze how these basic common elements of social medicine identified through the Latin American case are manifested in other historical currents within the field and in contemporary expressions of “protest medicine”. In these contexts, health professionals and volunteers provide care, document state violence, and transform medical testimony into political action. By situating these practices at the intersection of grassroots militancy and institutional incorporation, the article reflects on the enduring tensions and possibilities of social medicine as a global, evolving project oriented toward health equity and social justice.

## Introduction: Social medicine as a field with porous boundaries

Social Medicine is a field with porous boundaries. There is no single uncontested definition of social medicine, and the field frequently overlaps or coexists with neighboring disciplines, including medical anthropology, social studies of medicine and global health, and other related domains. As Anderson and colleagues have already stated, the field is “always elusive, always escaping precise definition and definitive realization” ([1], p.2). Moreover, a timely and significant volume on the Global Histories of Social Medicine presents multiple and contrasting understandings of the field, stressing its plurality across time, geography, and political systems [2]. Historically, social medicine spans reformist welfare-state initiatives and radical Marxist critiques, colonial hygiene campaigns and anti-imperialist struggles, institutionalized medical specialties and grassroots activism.

Scholars across different disciplines have attempted to address the problem of how to conceptualize social medicine, highlighting its plurality and the difficulty of stabilizing a common definition. Metaphors and concepts such as “avatar” [3], “trans-epistemic arena” [4], and “boundary object” [5], underscore that the field is both theoretically heterogeneous and historically situated. This contested landscape makes it relevant to revisit social medicine as a field. Rather than assuming conceptual coherence, we take the ongoing ambiguity itself as an empirical problem that requires examination. In this scenario, the pertinent question is not “-what is social medicine?”- but rather “-who has employed it, what has it achieved, and what possibilities does it enable”- (5, p.322), and how social medicine interacts with the broader global politics and ideologies. This feature is particularly significant, insofar as contemporary debates tend to emphasize porosity -and plasticity- at the expense of commonality [2].

Beyond these definitional debates, clarifying what social medicine offers is crucial because its analytical and political tools address problems faced by a wide range of practitioners, researchers, activists and policymakers involved. In a context of widening inequities, democratic erosion, and global crises, defining social medicine’s distinctive contribution helps readers understand why it continues to matter: it provides a coherent vocabulary and set of strategies for diagnosing structural drivers of ill-health and for acting upon them. Rather than a narrow disciplinary exercise, articulating the contours of social medicine equips diverse actors with conceptual, methodological, and political resources that can be mobilized in clinical practice, public health, community participation, and policy debates. Our analysis is based on a selective reading of the major scholarly debates that have historically defined the boundaries of social medicine.

The tension between SM’s constant evolution and the basic common elements that persist across its forms shows that not everything is possible. Not all research on medicine from the perspectives of the social sciences and humanities, nor the mere introduction of the social determinants approach, constitutes social medicine per se. While championing SM’s porous, polymorphous, and pragmatic nature, we argue — risking being labeled “demarcationists” — that it is essential to reflect on the few elements of a “common identity” that may be recognizable and translatable across sites.

We begin by comparing social medicine with some neighboring fields that are treated in some debates as interchangeable. To remain concise, we will briefly mention three fields: medical anthropology, social studies of medicine, and global health. We acknowledge the commonalities but stress that this resemblance obscures unique aspects of social medicine related to tackling health inequities and promoting social justice. We also explore the relationship between social medicine, community health/medicine, and preventive medicine. Then, and without a normative intention, we propose three elements that could constitute the basic common elements of social medicine, based on core tenets of Latin American Social Medicine (LASM) for this classification: 1) political commitment to social justice, 2) role of social sciences, and 3) participatory methodologies rooted in community participation. We draw on Latin American Social Medicine (LASM) as a particularly rich case, not only because of its historical depth and institutionalization since the 1960s, but also because it has often been overlooked in global debates despite its significant contributions. In the last section, we conclude by highlighting how the basic common elements of social medicine may appear either jointly or separately in other currents within the field and in contemporary expressions such as “protest medicine,” particularly in the context of uprisings in different countries.

## Social medicine and its neighboring disciplines

Social medicine has developed alongside several neighboring disciplines within the health and social sciences that share its interest in the relationship between society and health. While these fields often overlap in concerns and perspectives, each brings distinctive questions, methods, and goals. Examining the contrasts and convergences among them, particularly medical anthropology, social studies of medicine, and global health, helps to clarify what is unique about social medicine and why it matters today. These comparisons do not seek to essentialize or homogenize these fields; rather, they highlight dominant historical tendencies while acknowledging the internal diversity and critical traditions within each.

Medical anthropology and social medicine are neighboring disciplines, and both encompass a wide range of theoretical and applied approaches. Medical anthropology is a porous discipline with contested boundaries: while historically associated with the study of cultural meanings, practices, and experiences of health and illness, the field also includes strong critical and applied traditions. Medical anthropologists examine how beliefs and practices around health are shaped by cultural, political, historical, economic, and religious forces, and many engage directly with the structural and social determinants of health. Indeed, much of critical and applied medical anthropology closely parallels social medicine in its attention to inequality, power, and health system transformation [6–9]. Social medicine has its own intellectual history defined by the analysis and critique of the societal factors that contribute to or prevent illness. It analyzes how social and structural conditions (poverty, inequality, labor, racism, environment) shape population health and disease distribution. It asks how class, economy, and politics shape disease patterns, and how policy and health systems can reduce health inequities [10–12]. While more theoretical oriented MA provides ethnographical insight into lived experience and meaning of health and illness, applied medical anthropologists share SM’s commitment to transform health systems and social structures to reduce inequities. Applied and Critical Medical Anthropology (CMA) examine how broad socio-economic and political structures shape patterns of health, illness, and access to care at the level of everyday experience. CMA focuses on the political economy of health and keeps structural determinants and social justice at the center of global health explanations while promoting approaches that advance health equity and social justice [13].

Social Studies of Medicine (SSM) instead is an academic field that examines (bio)medicine as a social and cultural practice, a social institution and knowledge system, focusing on issues such as medical knowledge, technologies, professional power, patient-doctor interactions, and the politics of health and science [14,15]. Social medicine, in contrast, focuses on addressing health inequities by integrating social sciences with medicine to identify and act on “upstream” factors, the structural determinants of health, the reduction of health inequalities, and the strengthening of health systems. Both disciplines recognize the impact of social factors on health; the main difference between them relates to political engagement. SSM is largely critical but more academically detached. It investigates power relations, authority, and medicalization without necessarily proposing policy reform [16]. SM, on the contrary, is intrinsically reformist and political; it explicitly aims to transform social and structural conditions to reduce inequalities and support health as a social right, which has led in several countries to the creation of universal health systems [12]. It is the difference between a critical academia and bringing “academia to the ground” (“la cátedra a terreno”), as a social medicine initiative in Chile at the end of the 1950s defined, developing a program of integral medicine that enabled health professionals and students to work directly with neighbors and communities [17].

For its part, global health is an area for study, research, and practice that places a priority on improving health and achieving equity in health for all people worldwide. Social medicine has recently advanced several critiques of global health, questioning its top-down, technocratic, and donor-driven orientation that often neglects local contexts and disregards the structural determinants of health [18,19]. While global health targets specific diseases, risk factors, and measurable outcomes through technical interventions and cost-effective analysis, SM focuses on the structural and political determinants of health and disease and regards health as entangled with social justice and fights for rights [18]. The former relies on biomedical and epidemiological approaches to design and implement scalable interventions; the latter draws on critical social theories, critical epidemiology, community engagement, and participatory research to transform healthcare and achieve health equity. Global institutions and philanthropic foundations dominate global health, while grassroots movements, public sector action, and participatory politics drive social medicine [18,20,21]. Social medicine offers a critique of global health’s technocratic and depoliticized approach by emphasizing the political, economic, and historical roots of disease and the need for structural change to achieve health equity and social justice.

In some East Asian countries, such as South Korea and Japan, medical anthropology and the social study of medicine are often preferred over social medicine for examining medicine and healthcare from social science and humanities perspectives. This should be understood in relation to the historically limited institutional presence of social medicine in these contexts. After World War II, Japan developed its health system under strong U.S. guidance, emphasizing biomedical and technological approaches while structural critiques of capitalism and inequality remained less central. Universal coverage emerged primarily through an insurance-based model rather than through broad social movements for health equity, as occurred in parts of Latin America [22]. The US influence also contributed, according to some scholars, to the suspicion of social medicine, often associated with leftist and Marxist thinking [22]. A similar pattern has been described in South Korea, where related debates were partially incorporated into preventive medicine, a field oriented toward population health but generally less engaged with the broader social critique characteristic of social medicine. Health insurance, first introduced in South Korea in 1977 and extended to universal coverage by 1989, was expanded largely as part of state-led strategies for social stability rather than grassroots struggles for health rights [23]. In both Japan and Korea, medical education tended to prioritize biomedicine, clinical training, and health economics over political economy or social theory, although exceptions existed in specific academic settings. Unlike many Latin American experiences, where democratization struggles contributed to institutionalizing social medicine, health activism in Japan and Korea more often focused on insurance, labor, or environmental issues without consolidating a distinct social medicine framework [22–24].

Beyond the proximity and differences with medical anthropology, social studies of medicine and global health, community health/medicine, and preventive medicine have also been understood historically as close or proxy fields for social medicine in some contexts. One of the original sites of social medicine is related to community health in South Africa, the Community-Oriented Primary Care (COPC) of Pholela in what is today KwaZulu-Natal. Key social medicine figures such as Sydney and Emily Kark developed in Pholela in the 1940s and 1950s a community health experience which merged clinical care with public health and social action for a defined community, involving community diagnosis, multidisciplinary teams (physicians, community health workers, nurses and social workers) with active community participation to tackle the social determinants of health [25,26]. SM was criticized in Australia at the end of the 1970s for being too biomedical. The word “medicine” in social medicine excluded non-medical activists. “I think [social medicine] was a way for medicine to expand its view, and address social issues, and social concepts. It did privilege doctors of course, but that’s how things were at the time,” observes a leader of the community health movement in Sydney (cited in [27], p.249). SM was associated with medicalization, the move of the medical model into social settings. Community health seemed more appropriate, although there was also a convergence of both community health and SM, and later community medicine. However, as Anderson and his co-authors observe, “conceits and evasions such as ‘community medicine’ neither revived the political agenda of social medicine nor shored up physician authority.” Community medicine “tended to dwindle into a medical teaching and training stratagem” ([27], p.256).

In the United States, social medicine had to pass under the labels of preventive medicine and community health to be accepted: “Preventive medicine could cover the ground of social medicine without the baggage of socialized medicine” ([28], p.157). In contrast, Latin American reformers criticized preventive medicine for reducing structural determinants of health to individual risk factors, fostering individualism, and privileging biostatistics and epidemiology over social critique [29]. Sergio Arouca, a Brazilian physician, public health scholar, politician and one of the leading figures in Brazil’s health reform and the creation of the Unified Health System, famously called this the “preventivist dilemma” [30] and argued for a social medicine approach that foregrounds structural determinants as a path to transformative political change [31]. This critique also targeted community medicine, which was portrayed as a form of “medical policing” rooted in bourgeois ideology and sustaining the individualist orientation of preventive medicine [29,31]. We see how, in some sites, the morphing of social medicine into community health/medicine and or preventive medicine results in the depoliticization of health inequalities and the replacement of comprehensiveness in health by biological reductionism, as Arouca thought of preventive medicine.

Proponents of LASM avoid the concern that social medicine prioritizes medicine or doctors by shifting the emphasis from clinical practice to the social, cultural, economic and political determinants that define health and illness. Rather than stressing physicians’ authority, LASM conceives health is a collective and political project that overflows the medical realm and foregrounds inequality, violence, labor conditions, racism, and discrimination as key elements of the health-disease-care seeking phenomenon. Medicine becomes only one component within a broader project of social justice and community participation.

## Basic common elements of social medicine

In this section, we draw primarily on Latin American Social Medicine (LASM) to propose three core elements of social medicine that can be identified across its diverse historical and geographical expressions: 1) political commitment to social justice, 2) role of social sciences, and 3) participatory methodologies rooted in community participation. Although political commitment (Element 1) and participatory methodologies (Element 3) may seem similar, we distinguish them: the former refers to ideological orientations and forms of political action, while the latter concerns community-based practices through which social medicine has been operationalized.

We build on this section largely from LASM because it represents one of the most historically sustained, theoretically elaborated, and institutionally consolidated expressions of social medicine worldwide. Since the mid-twentieth century, LASM has generated a dense body of scholarship, influential national reforms, and community-based practices that explicitly link health to social inequality, political struggle, and democratic participation. Few other regional traditions combine theoretical innovation, long-term activism, and state-level institutionalization to the same extent. For this reason, LASM offers a particularly robust empirical and conceptual foundation from which to identify common elements that resonate across diverse histories and contemporary expressions of social medicine.

Our analysis adopts a historically grounded, comparative approach rather than a normative attempt to prescribe what social medicine should be, and the three basic common elements we identify reflect this analytical stance. We do not want to be prescriptive. These elements serve as guidelines for characterizing a movement, research area, or healthcare initiative as social medicine.

### 1. Political commitment to social justice and activism

What stands out in comparing social medicine with neighboring fields such as medical anthropology, social studies of medicine, and global health - and, in some contexts, with community health and preventive medicine - is its political commitment and transformative potential to address health inequities and advance social justice. We understand political in this context as the efforts aimed at transforming the structural determinants of health, expanding social rights, and reshaping institutions and power relations that produce inequality. We see this as a core element that social medicine has embodied in Latin America.

In Latin America, the first wave of SM (1920-1930s to late 1940s) carried a radical, revolutionary spirit, seeking broad social and political transformation. Yet, as Carter notes, once its early demands were institutionalized, they “led to complacency and ineffective bureaucratic routines,” provoking renewed critique and a second cycle of activism ([32], p.62). This second wave, emerging in the 1970s from disenchanted technocrats rather than revolutionaries, promoted SM’s incorporation into university programs and national health reforms.

Chile’s Servicio Nacional de Salud (SNS, 1952) represented the apex of the first wave, a “fluid field of political experiment” where diverse political actors converged around social justice and the socialization of medicine [32]. Brazil’s Sistema Único de Saúde (SUS, 1990) embodied the main achievement of SM’s revival from the mid-1960s to mid-1980s [4]. Both SNS and SUS translated the ideological commitments of social medicine into institutional reforms, demonstrating how state structures can become arenas for political struggle and redistributive transformation.

The institutionalization of social medicine distanced it from its revolutionary origins but yielded major achievements, including universal health systems, academic programs, professional associations, and the incorporation of reformers into state administration. Historically, many LASM traditions have sought transformation through the state, including legislative processes, public-sector reform, and alignment with broader left-wing and democratizing movements.

Over the last decade, however, health activism has undergone important shifts. Traditional movements tied to state institutions have concentrated on universal access, primary care strengthening, resistance to privatization, and social participation. Yet these agendas have been partially absorbed by the very institutions they sought to transform, limiting their disruptive potential. At the same time, new forms of activism -often situated outside formal health systems - have emerged in arenas such as the Latin American Association of Social Medicine (ALAMES) and the Brazilian Association of Collective Health (ABRASCO). These currents advance identity-based struggles around race, gender, and sexuality, signaling a reorientation of political action within social medicine and collective health [33].

### 2. The central role of social sciences

Social sciences are central to Latin American Social Medicine (LASM), shaping its theoretical framework and political strategies for health reform. Rather than auxiliary disciplines, they provide critical theory and participatory methods to analyze and transform health systems, reduce inequities, and promote social justice. LASM’s history is inseparable from the broader trajectory of social sciences in Latin America [31,32].

During the first wave of LASM (1920s--1940s), debates on the “social question” dominated movements in Peru, Costa Rica, Mexico, Chile, Brazil, and elsewhere. Most participants were medical doctors without formal social science training, influenced by positivism, eugenics, hygienism, and Catholic social doctrine [32]. The creation of the Economic Commission for Latin America and the Caribbean (CEPAL/ECLAC) in 1948 in Chile played a key role in developing dependency theory. Initially reformist, CEPAL introduced the structuralist center-periphery model and highlighted global inequalities. In the 1960s and 1970s, intellectuals such as Fernando Henrique Cardoso and Enzo Faletto, among others, radicalized the CEPAL framework and developed the dependency theory, arguing that underdevelopment and dependency were a necessary condition of capitalist development in the region [34]. LASM built on dependency theory to analyze underdevelopment, structural inequality, and imperialism as determinants of disease. The First Regional Meeting on Social Sciences Education in Medical Schools, held in Cuenca, Ecuador, in 1972 and organized by Juan César García from PAHO, marked a turning point. Participants rejected the prevailing functionalist approach as static, ahistorical, and detached from material conditions, advocating models linking health with production modes, socioeconomic context, and health system organization [31,35].

In the 1970s, LASM developed antifunctionalist, historical materialist analyses of health within a regional movement for the universal right to health. Drawing on Marxism, dependency theory, and liberation theology, scholars argued that economic growth and scientific progress often exacerbate health inequities as urbanization and industrialization deepen social disparities [36,37]. LASM critiqued the dominance of behavioral sciences in medical education for neglecting history and social structures, instead advocating frameworks grounded in social theory. Unlike public health or preventive medicine, LASM analyzed disease through categories such as social class, social reproduction, economic production, culture, ethnicity, and gender, aiming to “resocialize” health and illness [31]. Institutionally, García introduced social medicine courses in Brazil (1973) and Mexico (1974), with support from PAHO, and invited critical thinkers such as Ivan Illich and Michel Foucault [36]. The “preventivist dilemma” [30] revealed preventive medicine’s inability to address structural determinants.

In the same decade, LASM incorporated emerging theories across the social sciences, including structural Marxism, feminism, post-structuralism, and post-colonialism [32], as well as it integrated Latin American social theory, such as dependency theory, liberation theology, Freire’s critical pedagogy, and more recently, the philosophy of buen vivir— a decolonial health framework, originated outside LASM from indigenous movements and epistemologies, and incorporated into LASM’s broader decolonial turn. They all together helped to revitalize debates in social medicine and challenged functionalism, positivism, behaviorism, and developmentalism (desarrollismo) dominant in international health and development. These theories highlight that health and disease could only be understood through historically grounded categories such as social class, social reproduction, economic production, culture, ethnicity, and gender.

LASM scholars, because of these conceptual and methodological intersections, produced their own theoretical innovations, most notably critical social epidemiology. Developed by Jaime Breilh and Edmundo Granda in Ecuador, this approach linked health inequities to broader political-economic structures [11]. It was complemented by contributions from Cristina Laurell in Mexico and Naomar Almeida Filho in Brazil [11,31,38]. In this way, LASM combined the incorporation of external radical theories with the development and adaptation of internally generated concepts, collectively advancing a distinct, regionally grounded critique of health and society. More broadly, it contributed ongoing efforts to construct an “alternative” and “decolonial” health epistemology from the Global South [32].

A key innovation of LASM is replacing the Social Determinants of Health (SDH) approach promoted by the WHO with the LASM--Collective Health (LASM-CH) perspective, emphasizing the social determination of health-disease processes. Although elements of the SDH framework can support structural analysis, its dominant global formulation often depoliticizes inequalities, avoids references to capitalism and historical power relationship, and favors technocratic and governance-centered solutions. In contrast, LASM-CH explicitly criticizes capitalism and neoliberalism, views inequities as rooted in historical, political, and economic structures, and centers political struggle, collective action, and individuals as agents of change. In short, SDH is largely reformist and technocratic, whereas LASM is critical and transformative [10].

### 3. Participatory methodologies and community participation

Scholars and practitioners recognize LASM for its commitment to participatory and community-based methodologies, grounded in the conviction that health is socially determined and that communities must act as agents in defining and addressing health issues. In its early stages, LASM actors developed these methodologies through rich interactions with other critical disciplines, such as pedagogy and social work, framing them not only as scientific tools but also as political practices that democratized knowledge, empowered marginalized groups, and linked health to broader struggles for justice. For example, Paulo Freire’s Pedagogy of the Oppressed [39] was highly influential in social medicine and participatory research, introducing concepts such as dialogical praxis and critical consciousness [40].

Chile provided one of the first large-scale applications of participatory approaches since 1952. The National Health Service created neighborhood health committees where residents collaborated with health professionals to set priorities and monitor programs. These committees reflected the LASM principle that communities should not be passive recipients of services but co-creators of health strategies. Although the 1973 coup abruptly ended this experiment, the Chilean case demonstrated how participatory methodologies could be institutionalized in public systems, embedding community voices in decision-making within public systems [17].

Nicaragua, after the 1979 Sandinista Revolution, exemplified the attempt to institutionalize participatory health approaches at a national scale. The revolutionary government declared health a universal right and organized mass campaigns for vaccination, sanitation, and disease control through brigadistas drawn from schools, unions, and neighborhoods [41]. These volunteers embodied the LASM ethos of linking technical interventions with political education and collective mobilization. The campaigns, as Briggs and Mantini-Briggs [42] describe, sought not only to improve epidemiological outcomes but also to cultivate solidarity and critical awareness among participants. Despite the challenges of war and economic blockade, Nicaragua’s brigades remain a paradigmatic example of community-based participatory health in practice.

In Mexico, participatory practices grew around the work of community health promoters in Chiapas and Morelos during the 1980s. These promoters were trained in basic biomedical practices but also in methods of collective analysis that encouraged communities to link illness with poverty, labor exploitation, and political exclusion [43]. This approach turned health promotion into a two-way dialogue rather than a top-down transfer of information. Based on these experiences, Asa Cristina Laurell [38] argued that illness patterns are products of social structures rather than individual behaviors. The Mexican case illustrates how participatory methodologies were central to both grassroots empowerment and the development of a distinctive LASM theoretical framework.

Brazil offers one of the most institutionally consolidated experiences of community participation shaped by the broader process of democratization. Popular health movements in urban peripheries and rural areas in the 1970s and 1980s organized around access to water, sanitation, and medical care, using participatory diagnostics and assemblies to articulate demands. These grassroots practices converged with the movimento sanitário (health reform movement), a coalition of progressive professionals and activists who advocated universal, equitable, and participatory health care. Their combined efforts led to the creation of the Unified Health System (SUS) in 1990, enshrined in the 1988 Constitution, which guaranteed universal access and mandated participatory health councils at municipal, state, and national levels [44]. Brazil demonstrates how participatory methodologies, born in community struggles, could be institutionalized in state structures.

SUS emerged from the activism of professionals who were also political militants in the 1970s–1980s health reform movement [45]. Yet while many remain engaged in the system, their militancy has often been absorbed into bureaucratic and managerial logics, raising questions about its continuing oppositional and transformative force [46]. In Chile, similar tensions appear: health workers demonstrate strong loyalty to the public system, but this commitment is constrained by professional hierarchies and administrative pressures [47].

Citizen participation in Brazil is institutionalized through Health Councils at national, state, and municipal levels, with representation from both government and civil society [48,49]. National and local health conferences foster democratic debate and collective decision-making [50]. Within the Family Health Strategy (Brazilian primary care model), neighborhood meetings and local councils provide spaces for community feedback, while community health agents mediate between households and health teams [51]. Civil society organizations also engage in advocacy, monitoring, and accountability through public hearings, litigation, and forums [48].

Taken together, these cases show how participatory and community-based methodologies became central to LASM’s identity. They were shaped by interdisciplinary exchanges---such as the influence of Freire’s pedagogy---but more importantly by the lived practices of communities across Latin America. While many of these initiatives faced repression, resource constraints, or bureaucratic challenges, their legacy demonstrates the enduring relevance of participatory methodologies for confronting health inequities in the region [52]. At the same time, it should be noted that LASM has not had a monopoly on community participation; similar experiences emerged elsewhere, such as the Karks’ community health initiatives (Community-Oriented Primary Care – COPC) in Pholela in the 1940s and 1950s [25,26], the establishment of community health centers in Mississippi in the 1960s led by Jack Geiger [28], and community participation in rural primary healthcare in India in the 1950s and 1960s [3].

## Concluding remarks

Social medicine’s porous nature is evident not only in its conceptual plurality but also in its academic practices. In fact, much of the scholarship associated with social medicine is carried out by researchers in adjacent fields—such as medical anthropology, social studies of medicine, global health, preventive medicine, and community health—often surpassing the number of faculty formally appointed to social medicine and underscoring the field’s inherent fluidity. This permeability is visible in publication patterns: scholars working on social medicine frequently publish in journals primarily associated with these adjacent fields, rather than in journals explicitly dedicated to social medicine. Specialized social medicine journals are scarce, demonstrating the field’s cross-disciplinary orientation and the difficulty of containing it within rigid institutional boundaries.

This reality illustrates both a strength and a challenge of social medicine. On one hand, the field’s adaptability allows it to respond to diverse historical, geographic, and political contexts; on the other, it risks being conflated with other research traditions if its distinct focus on health inequities, community participation, social justice, and structural determinants of health is not clearly articulated. The field’s porosity, therefore, is not mere openness: it is a dynamic interface, a boundary zone where social medicine interacts with multiple epistemic and institutional domains, enriching both itself and the disciplines it engages with. This resonates with recent efforts to delineate the contemporary scope of SM [53], articulating shared critical and transformative pedagogical approaches across social medicine, collective health, and structural competency, expanding the field’s capacity to advance health as a human right.

In the same vein, the three elements we identify as the basic common elements of social medicine based on the Latin American case may appear either jointly or separately in other currents in the field during the twentieth century. For instance, SM in Scandinavia exhibits some frictions between political radicalism and institutionalization. During the 1930s, radical activists and leftist medical professionals wanted to reshape their societies, and SM contributed to the theoretical basis for the shift from capitalist to socialist societies. Many of those activists have later played important roles in defining health policies during the “golden age” of Scandinavian welfare systems (1940s-1970s). Thus, “the former revolutionary doctors had become nation builders; a medical expertise in the making of ‘the good society,’ and the capital invested to ‘safeguard the health of the people’ was not unproductive, rather ‘in the truest sense productive capital’” ([54], p.129).

In India, Gandhian revolutionary approaches were taken up by grassroots movements in the 1980s and 1990s and led to the rise of coalitions of political parties and leftist trade unions, among others, around issues of health and social rights. Such community health experiences were translated into national health policy with the introduction of the Community Health Workers (CHWs) program in the late 1970s [3]. In the US, the Black Panther Party and Latinx activism established in the 1960s and 1970s pursued radical visions of community health and social medicine, establishing free medical clinics and demanding health care that prioritized community needs. Such radical “bottom-up” community health campaigns and health revolutionaries in cities like NY faced the dilemma of relying on their own expertise or accepting allies from within the health system (white doctors) and carefully negotiating federal and state healthcare measures [28]. The situation is, however, different from other sites because the state did not play a central role in defining social medicine. Therefore, the oft-cited claim that social medicine is what emerges when a state fails to provide health to its people applies to some SM traditions, like the US, whereas in other traditions, like LASM, the state plays a central role in defining social medicine. SM is a public project to achieve health equity and social justice.

Beyond these traditions, protest medicine (PM) has emerged as a contemporary form of social medicine outside and sometimes against the state. It demonstrates how the three basic common elements of social medicine (political commitment to social justice, the central role of social sciences and participatory methodologies and community participation) materialize under conditions of acute social conflict. Although most volunteers and health professionals engaged in PM do not identify their actions as part of social medicine, the forms of practice that emerge in these contexts closely mirror social medicine’s ethical and analytical repertoire. PM therefore illustrates how social-medicine principles can reappear through practice, even without explicit theoretical lineage.

PM makes visible the political stakes of caregiving when the state fails to protect, or directly harms, its citizens. In Chile’s *estallido social*, mass demonstrations were met with unprecedented state violence during October 2019, producing nearly 4,000 injured protesters and over 400 cases of pellet-related ocular trauma. Volunteer medic brigades, formed by students, paramedics, and health professionals, mobilized spontaneously to provide first aid, triage, and evacuation in the streets [55,56]. Their presence constituted not only an emergency health response but also a public assertion of the right to safety, bodily integrity, and political participation. Similarly, in Lebanon, during the October 2019 protests, doctors and nurses treated injuries from riot-control weapons, denounced excessive force, and participated in labor strikes triggered by medicine shortages and the collapse of hospital financing, thus transforming clinical work into political engagement [57,58]. In Gaza, since October 2023, health workers have faced relentless bombardment, attacks on hospitals and ambulances, and critical shortages. Providing care under these conditions inevitably becomes a form of political commitment: a defense of life, humanitarian law, and civilian protection [59–61].

A second dimension of PM is the systematic production of medical evidence that enters human-rights investigations and international advocacy. In Chile, hospitals and clinicians documented injury patterns associated with crowd-control weapons, while volunteer brigades generated records that exposed the scale and modality of state violence, later used by oversight bodies and human rights organizations [55,56]. In Lebanon, clinical testimonies and case documentation became part of civil society reports on excessive force [57,58]. In Gaza, data collected by Palestinian providers, UN agencies, WHO, and NGOs on casualties, infrastructure destruction, and attacks on medical facilities have formed the backbone of humanitarian appeals and human-rights dossiers, even when structural reforms remain blocked [59–61]. PM thus reflects social medicine’s long-standing reliance on analysis, evidence, and documentation to illuminate structural determinants of harm.

PM also embodies participatory, community-embedded forms of organization. In Chile, volunteer brigades developed decentralized systems of first aid, evacuation, and logistical support integrated into protest spaces [55,56]. In Lebanon, field tents and makeshift care stations emerged as collective spaces where volunteers and professionals coordinated responses to unplanned mass gatherings [57,58]. In Gaza, community networks have become essential for sustaining care and navigation when formal operations are suspended due to insecurity [59–61]. These modes of collective action echo the participatory traditions of Latin American social medicine, from brigadistas and mutual aid initiatives to community-based diagnostics and collective health mobilization.

Taken together, PM offers a powerful contemporary illustration of social medicine’s common elements. It shows how political commitment, knowledge production, and participatory action reappear as practical necessities in contexts of conflict. PM thus underscores that social medicine is not only an institutional or academic tradition but a living project that resurfaces wherever communities mobilize to defend life, expose injustice, and confront health inequities.

We can see how SM historically and in its contemporary avatars oscillates between, on the one hand, the radical revolutionary spirit we find in grass roots and activist health movements that were at the base of LASM and other traditions such as the Scandinavian and Indian ones, as well as in contemporary forms protest medicine; and, on the other, the institutionalization of SM leading to the constitution of universal health systems, academic programs and professional associations. The latter has radically transformed political activism within the system with new forms of social participation (e.g., health councils) and of health activism outside the system.

Revisiting who has employed social medicine and what it has achieved, our analysis shows that the field has been mobilized by actors ranging from state reformers to grassroots activists and protest medicine brigades. Its key accomplishments—universal health systems, participatory community-based methodologies, and political movements for health equity—highlight both its transformative power and its internal tensions. The contemporary cases examined, including protest medicine, further demonstrate how social medicine expands public health possibilities by creating new political spaces, generating evidence on structural harm, and sustaining collective practices that protect life amid inequality and conflict.
